# Ear-Ache in Children

**Published:** 1883-08

**Authors:** 


### Ear-Ache in Children.

Dr. Samuel Sexton contributes an article on this subject to the *Medical Record*, May 5, 1883, in which he deprecates routine treatment or injections until we have found out the cause of the ache by careful examination, for in a very large number of ear-aches in childhood the causes are to be sought elsewhere than in the hearing organ itself, and they will be found to depend, for the most part, on nervous sympathy; the most prominent are dentition, dental caries, and colds in the head. Thus nervous impulses propagated from regions remote from the ear may give rise to pains in the ear—neuralgic otalgia—without perceptible hyperæmia; or the intensity of the congestion arising in a part so richly supplied with blood-vessels may manifest itself as an acute aural catarrh.

A GLUE ready for use is made by adding to any quantity of glue common whiskey instead of water. Put both together in a bottle, cork it tight, set aside for a few days, and it is ready for use. In cold weather it may have to be placed in hot water for a few minutes before it gets soft.

